# Supplementary material for: Analysis of chi angle distributions in free amino acids via multiplet fitting of proton scalar couplings
Source: Magn Reson (Gott). 2024 Aug 19;5(2):103–20. doi: 10.5194/mr-5-103-2024 (PMC11570886; doi:10.5194/mr-5-103-2024)
Supplement: The supplement related to this article is available online at: https://doi.org/10.5194/mr-5-103-2024-supplement. [file mr-5-103-2024-supplement.zip › Syed2024/data/fit1d_fitnmr_output.tar.gz!/fit1d/asp/400/bmse000031_simulation_1_nmredata/1H/sparse_1d.pdf]

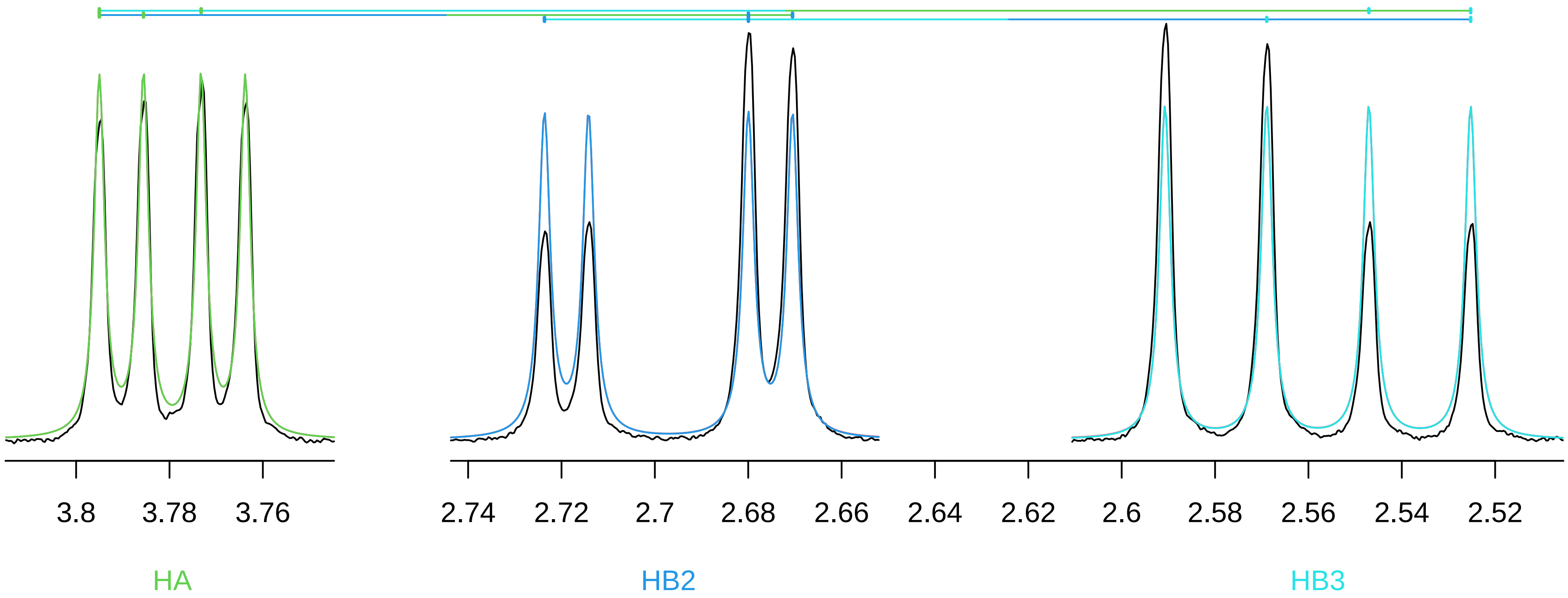
3.8
3.78
3.76
2.74
2.72
2.7
2.68
2.66
2.64
2.62
2.6
2.58
2.56
2.54
2.52
HA
HB2
HB3
